# A qualitative study of coach mentoring dentists in difficulty from a mentor perspective

**DOI:** 10.1038/s41415-024-8230-x

**Published:** 2025-05-09

**Authors:** Philip A. Gowers, David R. Radford, Carolina Machuca-Vargas, Chris Louca

**Affiliations:** 053830347835469467224 PhD Student, University of Portsmouth, United Kingdom; 638231334587303514879 Professor, University of Portsmouth Dental Academy, Faculty of Science and Health, University of Portsmouth, Portsmouth, PO1 2QG, United Kingdom; 996185939725634639643 Senior Lecturer in Dental Public Health and Research Lead, University of Portsmouth Dental Academy, Faculty of Science and Health, University of Portsmouth, Portsmouth, PO1 2QG, United Kingdom; 843154896910826032741 Professor, Director and Head, University of Portsmouth Dental Academy, Faculty of Science and Health, University of Portsmouth, Portsmouth, PO1 2QG, United Kingdom

## Abstract

**Introduction** The General Dental Council (GDC) regulates the United Kingdom (UK) dental profession and a dentist may be mandated to attend a fitness to practise (FtP) panel. In this study, such dentists are referred to as a ‘dentist in difficulty' (DD). Coach mentoring by a coach mentor (CM) is one of the GDC suggestions to support DDs to help them fulfil the conditions set by a FtP panel. There is little published literature on coach mentoring DDs.

**Aims** The aims of this study were to investigate the lived experiences of CMs, including their reasons for becoming a CM and their perceptions of both the DDs and the future of coach mentoring.

**Methods** Using a qualitative research methodology, 11 individual semi-structured interviews were undertaken with identified CMs (study participants). Recorded interviews were transcribed verbatim and analysed using thematic analysis to identify overarching themes.

**Results** Five themes and 21 subthemes were identified. All CMs reported that they offered confidential, effective and non-judgemental support for DDs. The CMs believed in the reactionary coach mentoring they delivered but considered that a more preventive approach should also be explored.

**Conclusion** CMs reported complex intrinsic and extrinsic factors affecting the DDs and felt that coach mentoring supports DDs to develop into safe, reflective and insightful practitioners.

**Supplementary Information:**

Zusatzmaterial online: Zu diesem Beitrag sind unter 10.1038/s41415-024-8230-x für autorisierte Leser zusätzliche Dateien abrufbar.

## Key points

- This research reported on the lived experience of coach mentoring a dentist in difficulty (DD) from a dental coach mentor (CM) perspective.
- CMs reported complex intrinsic and extrinsic factors affecting DDs and the support given to allow them to develop into safe, reflective, insightful practitioners.
- Further research is needed to investigate the perceptions of dentists in difficulty to elucidate the value of coach mentoring and how it can be used in a more preventive fashion, as well as the current reactionary approach.

**Supplementary Information:**

Zusatzmaterial online: Zu diesem Beitrag sind unter 10.1038/s41415-024-8230-x für autorisierte Leser zusätzliche Dateien abrufbar.

## Introduction

Dentists work in heavily regulated and stressful environments, with increasing reports of stress-related illnesses and burnout.^1^^,^^2^ The dental profession's regulator in the United Kingdom (UK) is the General Dental Council (GDC), whose primary purpose is to protect patient safety and maintain public confidence.^3^ Other UK regulatory bodies for dental practitioners include NHS England.^4^ The GDC maintains a register of registered qualified dental professionals, sets standards for the dental team, investigates complaints about a dental professional's fitness to practise (FtP) and work, and ensures the quality of dental education is maintained.^5^ All patient complaints to the GDC are investigated and the volume of FtP cases has varied over recent years, possibly due to the COVID-19 pandemic.^6^^,^^7^

**Fig. 1:**
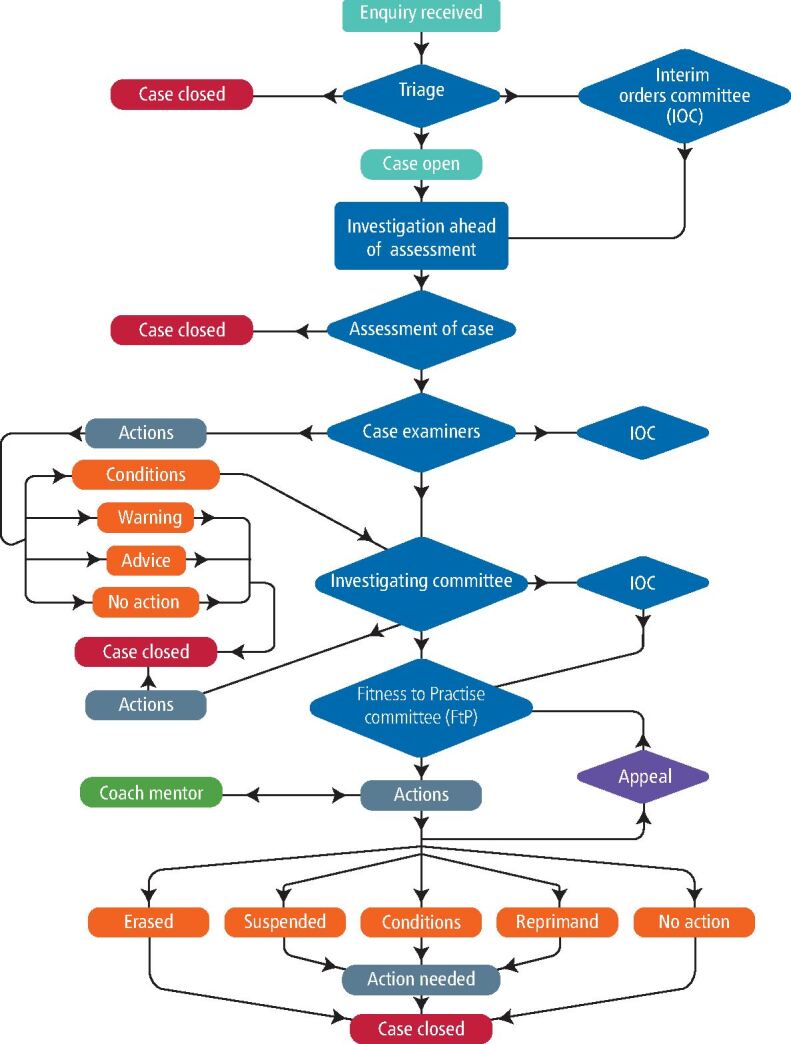
Summary of how the GDC FtP process functions

The process of how the GDC manages the dental profession can exacerbate a dentist's stress and potential for burnout.^8^^,^^9^^,^^10^ This often occurs following an upheld patient complaint, when a dentist may be mandated to attend an FtP panel (see Fig. 1).^11^ A dentist attending an FtP panel is often described as a ‘dentist in difficulty' (DD). Once a DD has agreed to the conditions imposed upon them by an FtP panel, they are advised by the GDC to find a suitable dental professional to support them, including a coach mentor (CM) (not mandatory) who will monitor and report on their progress, as they remediate the issues identified. DDs self-fund this support (see Fig. 1).^12^^,^^13^

All dentists with GDC-applied conditions, reprimands, or a ‘no action required' status can continue to practise dentistry but those erased or suspended cannot.^13^^.^^14^

Few dentists have experienced an FtP panel and most are unaware of what help is available to move forward. There is a wide range of available support for a DD (Table 1).^14^

**Table 1:** Organisations that play a supporting role for dental professionals in difficulty^14^

**Specialist indemnity providers**	
British Dental Association Indemnity	www.bda.org/indemnity
Dental Protection Ltd	www.dentalprotection.org
Dental Defence Union	www.theddu.com
Medical and Dental Defence Union of Scotland	www.mddus.com
**Educational support**	
NHS Scotland, Training, Revision and Mentoring Support Programme	TRaMS@nes.scot.nhs.uk
NHS Wales Dental Deanery	https://heiw.nhs.wales/education-and-training/dental/
Northern Ireland Medical and Dental Training Agency	www.nimdta.gov.uk
Committee of Postgraduate Dental Deans and Directors (COPDEND)	www.copdend.org
NHS England Workforce, Training and Education	www.hee.nhs.uk
**Health support**	
Practitioner Health Programme	www.php.net
Dentists' Health Support Programme	www.dentistshealthsupporttrust.org
British Doctors and Dentists Group	www.bddg.org
Sick Doctors Trust	www.sick-doctors-trust.co.uk
Practitioner Health	www.practitionerhealth.nhs.uk
**Mentoring/professional support**	
Local Dental Committees	www.bda.org/about-us/our-structure/representative-committees/general-dental-practice/local-dental-committees/
Dental Mentors UK	www.dentalmentorsuk.com
**Advisory support**	
NHS Resolution	www.resolution.nhs.uk
NHS Scotland	www.gov.scot
NHS Wales	www.wales.nhs.uk
NHS England	www.england.nhs.uk
**Professional organisations**	
British Dental Association including Indemnity and Benevolent Fund	www.bda.org
British Association of Dental Nurses	www.badn.org.uk
British Society of Dental Hygiene and Therapy	www.bsdht.org.uk
Dental Technologists Association	www.dta-uk.org
British Dental Industry Association	www.bdia.org.uk
National Association of Dental Advisers	www.nada-uk.org
British Association of Dental Therapists	www.badt.org.uk
Orthodontic National Group	www.orthodontic-ong.org
British Association of Clinical Dental Technology	www.bacdt.org.uk

DDs have complex issues, which include intrinsic factors (eg stress and burnout, poor health, and impaired performance) and extrinsic factors (eg culture, patient interactions, and poor work-life balance).^14^ DDs complex issues are often associated with mental health issues.^15^^,^^16^

DDs going through an FtP panel are subject to conditions designed to allow them to develop into a safe and reflective dentist.^17^^,^^18^ The GDC has suggested coach mentoring as support for DDs to continue being registered dentists (see Fig. 1).^14^ The CM's role is to guide the DD to accept that there may be other ways of thinking and to be honest with themselves about how a significant event may have affected them, as an individual both personally and professionally.^19^ The DD needs to work through the outcomes of the FtP panel through reflection by challenging pre-existing assumptions with critical analysis in a confidential, safe and non-judgemental environment.^20^ Other than some anecdotal evidence, there is little published literature on coach mentoring DDs in the UK and worldwide (online Supplementary Figure 1). This study aimed to investigate the lived experiences of CMs, including their reasons for becoming a CM and their perceptions of both the DDs and the future of coach mentoring.

## Methods

Using a qualitative research methodology, the study explored the lived experiences of coach mentoring a DD from a CM's perspective. Research ethics committee approval was granted by the University of Portsmouth Faculty of Science and Health Ethics Committee (Ref No: SHFEC 2022-088). Semi-structured video conference interviews of 11 experienced CMs (study participants), known to the primary researcher, were undertaken between February and March 2023. ^21^ Study participants were identified using a snowball sampling technique whereby CMs identified other CMs willing to participate in the study.^22^ CM participants included in this study were all experienced (qualified over ten years), GDC-registered dentists and had GDC-acceptable levels of coach mentoring experience. They had all supported at least one DD to GDC standards. CM participants were excluded if they did not meet the inclusion criteria. Due to the small number of dental CMs in the UK, to maintain anonymity, no participant demographic data were collected (eg age, sex, number of years qualified, specialist/non-specialist practice).

All CM participants gave their full consent to participate in the study and all interviews were conducted remotely via Zoom video conferencing at a time of the participant's choosing. Interviews were recorded, transcribed verbatim and stored on a password-protected Google Drive. Each transcript was sent to the respective CM participant to check the accuracy of the transcribed interview and subsequently anonymised. All CM participants agreed with their transcriptions and manuscripts. A six-phase coding framework for thematic analysis was used to identify themes and patterns in the data.^23^ The phases are: familiarisation of data; generation of codes; combining codes into themes; reviewing themes; determining the significance of themes; and reporting findings. QSR NVivo data analysis software (Version 12, QSR International, Warrington, UK) was used to manage and analyse the data collected from the interview transcripts. Overarching themes were developed independently by two of the researchers (PAG and DRR) with reference to the primary aims of the study. The primary researcher was an insider researcher, who had experienced many of the same situations, events, issues and problems as the CM participants. This gave researcher PAG the ability to draw upon understanding and experience when asking questions or while probing in the interviews. The researchers acknowledged and considered this reflexivity bias.^24^ Subthemes were clustered under the main themes. The transcripts were subsequently re-read, and themes and subthemes were refined and confirmed by a series of four round table discussions between the two researchers. Both researchers acted independently and then came together to agree on the themes and the agreed themes were then included in the analysis. PAG is a former general dental practitioner who has been a qualified advanced CM for 12 years. DRR is an experienced qualitative researcher and senior academic at the University of Portsmouth. Personal biases and assumptions that may have affected how the research was conducted were reflected upon, acknowledging insider bias during the formulation of the research questions, data analysis and drawing up of conclusions.

## Results

**Fig. 2:**
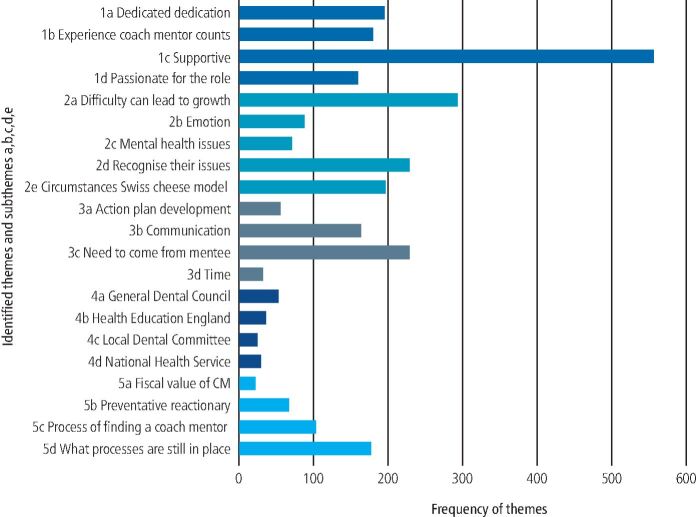
Thematic analysis of the CM participants' transcripts showing identified themes and sub-themes (Y) and frequency of occurrence (X)

The results are presented as five themes: 1) who are the CMs; 2) CM perceptions of the mentees; 3) the coach mentoring process; 4) the regulator and regulations; and 5) the future, and 21 subthemes (Fig. 2).

### Theme 1: The coach mentors

Theme one focused on the CM participants' formative experiences as practitioners and their reasons for becoming coach mentors:

- ‘*We were fortunate enough, perhaps, to have been mentored, even though the term was not used at the time, so perhaps an experienced colleague would take us under their wing, show us the ropes, be available if something went a little awry*' (participant 11).

This allowed a natural progression for them to become coach mentors:

- ‘*I was given the opportunity to develop my skills in a different way. So, it was for me. It was just a natural developmental option I've always been interested in helping dentists in difficulty*' (participant 3).

Four subthemes were identified, discussed below.

#### Theme 1(a): Dedication

The participants were dedicated and showed great commitment to the dental profession:

- ‘*There's a wealth of experience of helping and supporting individuals in a very positive way. For the benefit of the profession as a whole'* (participant 10).

#### Theme 1(b): Experience

The participants all reflected that they were experienced practitioners but as they developed, so did their coach mentoring skills:

- ‘*A CM is not there to solve all the problems, but it is to give that individual the ability to see where they've got to change and develop. The experience allows each DD to develop at their own pace and style'* (participant 8).

#### Theme 1(c): Supportive

This was the most common theme and all participants were supportive of their mentees to fulfil the conditions set by the FtP panel:

- ‘*They had very little appreciation for these huge skills that they had as a dentist that were way beyond the practical stuff of working with a patient, and I still feel that is an aspect of mentoring and coaching that's massively underdeveloped. With that support around them and people still believing in them, they can overcome and they can remediate and get back to living full lives and being good dentists'* (participant 11).

#### Theme 1(d): Passionate for the role

One of the most common characteristics of CMs was that they were passionate about coach mentoring:

- ‘*But for the grace of God…we never know what we might be facing, and these individuals, for whatever reason they've got themselves in a pickle…with support around them, that's ultimately what it's all about, and we shouldn't have a society that just writes people off'* (participant 8).

### Theme 2: The CM perception of their mentees

Theme two focused on the CM participants' perception of their DDs.

Five subthemes were identified, discussed below.

#### Theme 2(a): Difficulty can lead to growth

Dentists can find themselves in difficulty and the role and value of coach mentoring is to support and develop them through this difficulty:

- ‘*You're going to feel good that this has happened to you. The majority learn when things go wrong - when things go right, you don't learn'* (participant 7).

#### Theme 2(b): Emotion

The participants responded by recognising that DDs are highly stressed and emotional:

- ‘*Registrants who are in difficulty need emotional support, either angry, guilty and/or ashamed, and they need somebody to listen to their story, because along the way they have had very little control'* (participant 4).

#### Theme 2(c): Mental health issues

Mental health issues were reported as common among dentists going through this process:

- ‘*It's incredibly stressful, it's important to have somebody who can help you to maintain your mental health during that time'* (participant 1).

#### Theme 2(d): Need to come to a place to recognise their issues

Mentees need to come to a place to recognise their issues and a CM can help them to reflect on their position, but it must be mentee-led:

- ‘*It's not a punishment, but it is an opportunity to stop and then reflect on why you're in this situation. Our job as a CM is very much to help them get to that point of being able to accept what's happened, whatever it is, to use the complaint or the difficulty as a positive step but the mentee must want to change'* (participant 9).

#### Theme 2(e): Circumstances; Swiss cheese model

Participants reported that DDs sometimes, due to their circumstances, fall through the holes:

- ‘*Then they've forgotten to deal with it, or they're overwhelmed, and it's like the Swiss cheese theory that sadly the holes rather than the stars align, and they fall through. Things have just snowballed out of control for them'* (participant 4).

### Theme 3: The process

Theme three identified the process of coach mentoring a DD and how the CM participants used their coach mentoring skills and techniques to support a DD.

Four subthemes were identified, discussed below.

#### Theme 3(a): Action plan

Most participants used an action plan to help develop a DD within a defined structure:

- ‘*An action plan you have to which you develop with them, to have their goals set or it would just be open-ended'* (participant 5).

#### Theme 3(b): Communication

How well a mentee communicates was identified as a very important part of their success:

- ‘*Often dentists in difficulty do not have the right ability to communicate, not necessarily with everyone, but with some people particularly when something does go wrong'* (participant 10).

#### Theme 3(c): Need to come from mentees

Fundamentally, the need must come from the mentee for the CM participant to be able to support the mentee:

- ‘*If someone is willing to take help, then actions become easier. But when I've spoken to someone who's in total denial, I can't push someone for help, if not willing to accept help, or who is not even willing to accept that there is a problem*' (participant 6).

#### Theme 3(d): Time

The mentees needed an individual, time-limited contract because the regulators have a timeframe which the mentees are required to follow. The CM participants were always aware of the associated financial cost, as were their mentees:

- ‘*It just took time. The time limit is usually set by the GDC hearings. But time is money and money to somebody who's paying for a service'* (participant 4).

### Theme 4: The regulator and regulations

The fourth theme was the impact of the regulators and how they enforced the regulations.

Four subthemes were identified, discussed below.

#### Theme 4(a): GDC

CM participants felt they supported the mentees very well when involved with the GDC FtP panel:

- ‘*A lot more of them would have a difficult time with their GDC cases. Some of them would have much harsher sanctions. Some of them would be in a bad place*' (participant 11).

#### Theme 4(b): Health Education England

Health Education England supported DDs by offering coach mentoring, but when the organisation changed to NHS England Workforce, Training and Education, coach mentoring was no longer offered:

- *‘Universal coach mentoring was offered within our regulated Health Education England deaneries but has now stopped*' (participant 4).

#### Theme 4(c): Local Dental Committee support of DDs

- ‘*It would be through the LDC [local dental committee] PASS [Practitioner Advice Support Scheme] scheme and the panel would direct the dentists in difficulty to the contacts at the LDC PASS team so it's a very direct referral'* (participant 5).

#### Theme 4(d): National Health Service

NHS (National Health Service) dental teams regulate dental contracts and dentists with NHS performer numbers. The NHS can impose conditions on a dentist with an NHS performer number as well as the GDC:

- ‘*NHS England getting involved, which has heaped the pressure on them, which has then made the whole situation worse. And in effect, some of them got to the point where they couldn't function'* (participant 11*).*

### Theme 5: The future

Theme five focused on the future of coach mentoring.

Four subthemes were identified, discussed below.

#### Theme 5(a): The fiscal value of coach mentoring

- ‘*They are mostly keen and happy to spend really quite large sums of money on implants or aesthetic dentistry and believe it's a good investment. Yet the best investment is to learn how to mentor and be mentored because they'll use that across their career'* (participant 11).

#### Theme 5(b): Preventive and/or reactionary

Participants highlighted that some dentists seek CMs as part of a reactionary process following a regulator-led suggestion to find one. However, CM participants stated that a preventive approach would be more desirable:

- ‘*Quite a few dentists are not in trouble with the regulator but they are concerned that they may get into trouble with the regulator in future. They look at coach mentoring as a preventive aspect of their career'* (participant 3).

#### Theme 5(c): Process of finding a coach mentor

CM participants stated that the process of finding a CM has changed:

- ‘*DDs have had to find their own support. There are a few groups that they can find online and defence indemnity firms. More local areas are starting to reinstate or reinvigorate their LDC PASS schemes to support dentists'* (participant 10).

#### Theme 5(d): What processes are in place

CM participants had varied knowledge of the processes that are still in place for a DD to find a CM:

- ‘*NHS England panel would direct through the LDC PASS scheme. The GDC has only just moved to this ‘development advisor role' and if asked, will direct DDs to about four bodies that provide dental CMs for GDC performance issues'* (participant 5).

## Discussion

This study investigated the lived experiences of CM participants, including their perceptions of the dentists in difficulty under their care and support. This research showed that the CM participants perceived that coach mentoring can play a valuable role in supporting a DD to fulfil their FtP conditions, by gaining self-awareness and reflection.^25^^,^^26^^,^^27^ The impact of coach mentoring on the mentees was compared by some CMs to the ‘Kintsugi effect' of broken pottery being repaired with gold; coach mentoring can play a valuable role in helping dentists in difficulty to gain self-awareness and reflection. This is the gold that nurtures our colleagues.^28^^,^^29^ It is evident from this study that the reasons CM participants became CMs were because they are supportive, experienced, compassionate dentists who had been mentored in their own careers and trained to become CMs with support and empathy.

The CM participants perceived that the DDs had highly complex intrinsic and extrinsic factors working together, and often displayed deep emotions, such as shame, guilt and anger. CM participants said DDs can find themselves isolated and feel abandoned.

The present study highlights the importance of a structured action plan to allow the mentee to develop, with the help of a CM, thereby satisfying the conditions imposed by the GDC so that the DD can continue to work clinically. CM participants commonly identified the preventive role of good communication with patients resulting in improved patient satisfaction. CM participants confirmed that for coach mentoring to succeed, the mentees needed to be engaged with the process. Coach mentoring a DD must be completed within a defined timeline dictated by the FtP conditions; however, some CM participants continued to support their mentee afterwards on an individual basis. Although the GDC acknowledges the role of coach mentoring for the profession, it is not a mandatory requirement and DDs need to be self-funded. DDs may choose to go through a disciplinary process without any CM support.^30^

Although this study has explored the experiences of coach mentoring by the CM participants, they commonly reported the desirability of a more preventive rather than reactionary approach, beginning at the undergraduate level and progressing beyond graduation.^31^ All the CM participants expressed concerns about the future, particularly the lack of funding for training new CMs.

The limitations of this research include the limited number of CMs interviewed and that the primary researcher was an insider researcher.^21^^,^^24^ As a caring profession, dental professionals need to become more compassionate towards their colleagues.^25^^,^^26^ The researchers acknowledge that the perceptions DDs have of coach mentoring are unknown. Further research is needed to investigate the perceptions of DDs to elucidate the value of coach mentoring and how it can be used in a more preventive fashion, as well as the current reactionary approach.

## Conclusion

Within the limitations of the study, the main reason for CM participants to become a CM were identified. The CM participants were supportive of their mentee DDs and nurtured their development into safe, reflective and insightful practitioners. All CM participants identified a complex interaction of intrinsic factors (for example mental health issues and burnout) and extrinsic factors (such as toxic work environments and poor work-life balance) from the DD. CM participants also recognised their limitations and sign-posted their mentees to other sources of support. The future of coach mentoring is unknown due to uncertainties regarding the training and validation of future CMs.

## Supplementary Information

Supplementary Figure 1 (PDF 174KB)

## Data Availability

The authors confirm that the data from which the findings of this study are derived are available within the references and/or its supplementary materials.
