# Supplementary material for: A qualitative study of coach mentoring dentists in difficulty from a mentor perspective
Source: Br Dent J. 2025 May 9;238(9):720–5. doi: 10.1038/s41415-024-8230-x (PMC12064435; doi:10.1038/s41415-024-8230-x)
Supplement: Supplementary file 1 — Supplementary Figure 1 (PDF 174KB) [file 41415_2024_8230_MOESM1_ESM.pdf]

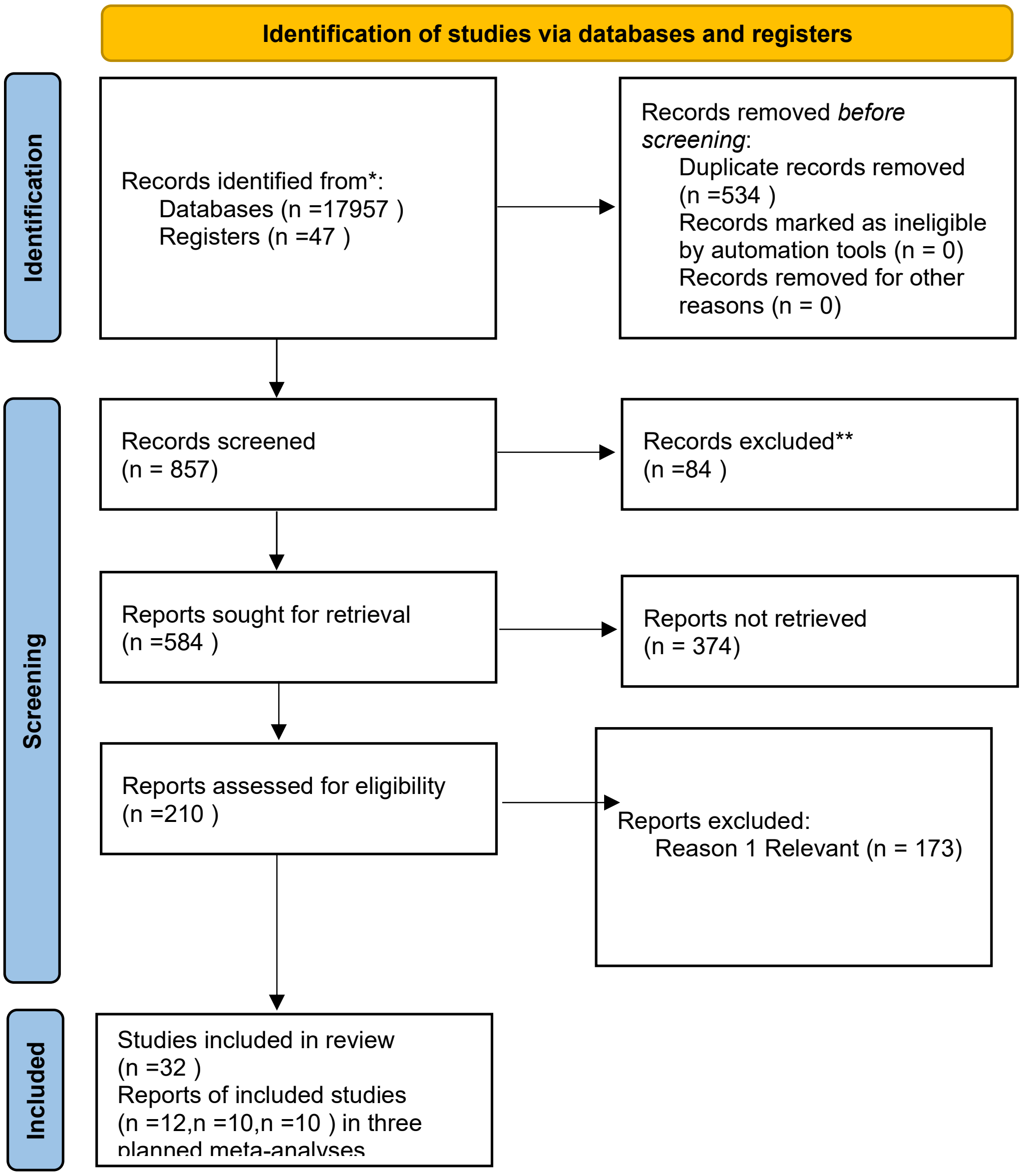

*Consider, if feasible to do so, reporting the number of records identified from each database or register searched (rather than the total number across all databases/registers).

**If automation tools were used, indicate how many records were excluded by a human and how many were excluded by automation tools.
